# The Establishment of New Thresholds for PLND-Validated Clinical Nomograms to Predict Non-Regional Lymph Node Metastases: Using ^68^Ga-PSMA PET/CT as References

**DOI:** 10.3389/fonc.2021.658669

**Published:** 2021-04-15

**Authors:** Jianhua Jiao, Zhiyong Quan, Jingliang Zhang, Weihong Wen, Jun Qin, Lijun Yang, Ping Meng, Yuming Jing, Shuaijun Ma, Peng Wu, Donghui Han, Andrew A. Davis, Jing Ren, Xiaojian Yang, Fei Kang, Qiang Zhang, Jing Wang, Weijun Qin

**Affiliations:** ^1^ Department of Urology, Xijing Hospital, Fourth Military Medical University, Xi’an, China; ^2^ Department of Nuclear Medicine, Xijing Hospital, Fourth Military Medical University, Xi’an, China; ^3^ Institute of Medical Research, Northwestern Polytechnical University, Xi’an, China; ^4^ Department of Medicine, Division of Hematology/Oncology, Robert H. Lurie Comprehensive Cancer Center, Feinberg School of Medicine, Northwestern University, Chicago, IL, United States; ^5^ Department of Radiology, Xijing Hospital, Fourth Military Medical University, Xi’an, China

**Keywords:** prostate cancer, PET/CT, distant, lymph node metastases, PSMA, impact, nomogram

## Abstract

**Purpose:**

PLND (pelvic lymph node dissection)-validated nomograms are widely accepted clinical tools to determine the necessity of PLND by predicting the metastasis of lymph nodes (LNMs) in pelvic region. However, these nomograms are in lacking of a threshold to predict the metastasis of extrareolar lymph nodes beyond pelvic region, which is not suitable for PLND. The aim of this study is to evaluate a threshold can be set for current clinical PLND-validated nomograms to predict extrareolar LN metastases beyond pelvic region in high-risk prostate cancer patients, by using ^68^Ga-PSMA PET/CT as a reference to determine LN metastases (LNMs).

**Experimental Design:**

We performed a retrospective analysis of 57 high-risk treatment-naïve PC patients in a large tertiary care hospital in China who underwent ^68^Ga-PSMA-617 PET/CT imaging. LNMs was detected by ^68^Ga-PSMA-617 PET/CT and further determined by imaging follow-up after anti-androgen therapy. The pattern of LN metastatic spread of PC patients were evaluated and analyzed. The impact of ^68^Ga-PSMA PET/CT on clinical decisions based on three clinical PLND-validated nomograms (Briganti, Memorial Sloan Kettering Cancer Center, Winter) were evaluated by a multidisciplinary prostate cancer therapy team. The diagnostic performance and the threshold of these nomograms in predicting extrareolar LNMs metastasis were evaluated *via* receiver operating characteristic (ROC) curve analysis.

**Results:**

LNMs were observed in 49.1% of the patients by ^68^Ga-PSMA PET/CT, among which 65.5% of LNMs were pelvic-regional and 34.5% of LNMs were observed in extrareolar sites (52.1% of these were located above the diaphragm). The Briganti, MSKCC and Winter nomograms showed that 70.2%-71.9% of the patients in this study need to receive ePLND according to the EAU and NCCN guidelines. The LN staging information obtained from ^68^Ga-PSMA PET/CT would have led to changes of planned management in 70.2% of these patients, including therapy modality changes in 21.1% of the patients, which were mainly due to newly detected non-regional LNMs. The thresholds of nomograms to predict non-regional LNMs were between 64% and 75%. The PC patients with a score >64% in Briganti nomogram, a score >75% in MSKCC nomogram and a score >67% in Winter nomogram were more likely to have non-regional LNMs. The AUCs (Area under curves) of the clinical nomograms (Briganti, MSKCC and Winter) in predicting non-regional LNMs were 0.816, 0.830 and 0.793, respectively.

**Conclusions:**

By using ^68^Ga-PSMA PET/CT as reference of LNM, the PLND-validated clinical nomograms can not only predict regional LNMs, but also predict non-regional LNMs. The additional information from ^68^Ga-PSMA PET/CT may provide added benefit to nomograms-based clinical decision-making in more than two-thirds of patients for reducing unnecessary PLND. We focused on that a threshold can be set for current clinical PLND-validated nomograms to predict extrareolar LN metastases with an AUC accuracy of about 80% after optimizing the simple nomograms which may help to improve the efficiency for PC therapy significantly in clinical practice.

## Highlights

^68^Ga-PSMA PET/CT demonstrated that nearly one-third of LNMs in high-risk treatment-naïve PC patients were non-regional and the new information obtained from whole-body ^68^Ga-PSMA PET/CT has the potential to benefit the nomograms-based clinical decision-making in more than two-thirds of the patients, leading to more personalized treatment. ^68^Ga-PSMA PET/CT can be used to exclude patients with non-regional lymph node metastases (LNMs) before pelvic lymph node dissection (PLND) in order to improve the efficiency of prostate cancer therapy in clinical practice. With a higher cutoff value, the clinical nomograms have the potential to predict non-regional LNMs as well.

## Introduction

Prostate cancer (PC) is the most commonly diagnosed malignant tumor and the second leading cause of cancer associated death in men worldwide (1). The most common route of metastasis is lymphogenous spread (2), and regional lymph node metastases (LNMs) can be observed in high-risk PC patients at 19.4% (3). The situation of LNMs can greatly influence clinical decision making in PC patients. Currently, the conventional imaging techniques such as computed tomography (CT) and multiparametric magnetic resonance imaging (mpMRI) had limited utility because of their relatively low sensitivities of approximately 40.0% for detection of LNMs (4). To better predict the risk of regional LNMs in PC patients, multiple pelvic lymph node dissection (PLND) validated nomograms, such as the Briganti, Memorial Sloan Kettering Cancer Center (MSKCC) and Winter nomograms were created to identify optimal candidates for PLND in treatment-naïve PC patients, according to EAU (European Association of Urology) and NCCN (National Comprehensive Cancer Network) guidelines (5–7). PLND should be performed in the patients with LNMs risk higher than 2% in MSKCC nomogram, 5% in Briganti nomogram or 7% in Winter nomogram (5–7).

The incidence rate of non-regional LNMs leading to unnecessary PLND in treatment-naïve PC patients were as high as 36.0% (8). However, according to the EAU–ESTRO–SIOG and NCCN guidelines, there is no specific examination for detecting non-regional LNMs in guidelines and only a cross-sectional abdominopelvic imaging (CT/MRI) and a bone scan are routinely recommended for staging purposes (7, 9). Therefore, many PC patients may receive unnecessary PLND because they already have non-regional LNMs. There were no thresholds in PLND-validated clinical nomograms (MSKCC, Briganti and Winter) to exclude the PC patients with non-regional LNMs. If there were thresholds to identify the PC patients with non-regional LNMs in PLND-validated clinical nomograms, the nomograms can be better used in selection of PLND candidates.

However, the detection of non-regional LNMs and development of nomograms to predict non-regional LNMs were limited by the imaging techniques to detect non-regional LNMs. In clinical practice, there was no routine imaging examination for non-regional LNMs and the nodal staging largely depended on preoperative pelvic CT/MRI or histopathological results from PLND. The whole-body MRI and biopsies for non-regional LNMs were hard to performed routinely. With high accuracy, ^68^Ga-PSMA PET/CT can be an effective imaging modality to detect non-regional LNMs. Prostate-specific membrane antigen (PSMA), a type II transmembrane protein, was overexpressed in 98.0% of PC associated LNMs (10). ^68^Ga-labeled prostate-specific membrane antigen PET/CT (^68^Ga-PSMA PET/CT) is a novel molecular imaging technique with promise compared to conventional imaging techniques for detection of LNMs for primary staging of PC patients (4, 11, 12). The use of ^68^Ga-PSMA PET in probing regional LNMs of PC was validated in a series of prior studies (13–16). The specificity of ^68^Ga-PSMA PET for detecting regional LNMs ranged from 80.0% to 100.0% (13) and confirmed high specificity of over 95.0% in large cohorts (17, 18). In hence, ^68^Ga-PSMA PET is now an established imaging technique to improve the detection of non-regional LNMs in prostate cancer (19).

The disease with regional LNMs can be considered as locoregional progressive disease between localized disease and the oligometastatic disease or systemic metastatic disease with distant LNMs (20, 21). Therefore, the aim of the present study was to investigate whether clinical nomograms (Briganti, MSKCC and Winter) can predict non-regional LNMs and to generate cutoff values to predict distant LNMs. Further, we analyzed the potential added benefit of visualizing newly detected distant LNMs by ^68^Ga-PSMA PET/CT to existing nomograms-based clinical decision-making. The non-regional LNMs observed by ^68^Ga-PSMA PET/CT were detected and monitored by multiple imaging techniques in follow-ups of the patients. In addition, we depicted metastatic pattern of LNMs in high-risk treatment-naïve PC patients.

## Patients and Methods

### Patients and Study Design

We retrospectively reviewed a database from a large tertiary care hospital in China for patients with pathologically confirmed PC who underwent ^68^Ga-PSMA PET/CT from April 2017 to October 2019. Patients were included in the study if they met the following inclusion criteria: (1) transrectal ultrasound (TRUS)-guided 12-core biopsy to pathologically confirm PC; (2) Gleason score (GS); (3) clinical tumor stage; (4) pretreatment total prostate specific antigen (tPSA). Patients were excluded if they received any treatment before ^68^Ga-PSMA PET/CT, such as androgen deprivation treatment (ADT), radical prostatectomy (RP), radiotherapy (RT), or chemotherapy. Other exclusion criteria included patients with negative PSMA expression on primary PC tumor validated by immunohistochemistry (IHC), and patients who had an interval between tPSA data and ^68^Ga-PSMA PET/CT that was more than 30 days. All patients had a bone scan and an mpMRI as well as ^68^Ga-PSMA PET/CT. Ultimately, a total of 57 patients with sufficient clinical data were eligible for analysis. Mean patient age was 69.4 ± 8.2 years (Median 68.5, range 40-84) and the mean serum PSA at imaging was 283.9 ng/ml (median 28.91, range 0.09-8447). All patients had high risk PC, according to the D’Amico standard (22).

This study was performed in the Urology Department and Nuclear Medicine Department of the Fourth Military Medical University Affiliated Hospital (Xijing Hospital, Xi’an, Shaanxi, China). The study was approved by the Ethics Committee of Fourth Military Medical University, and all participating patients provided written informed consent. The research was conducted in accordance with the Declaration of Helsinki and national regulations. Deidentified data were collected in a central database at Fourth Military Medical University.

### Histological Examination

A TRUS-guided 12-core prostate biopsy with necessary additional target biopsy was performed for each patient’s biopsy. All resected tissue of primary PC tumors and lymph nodes from surgeries were formalin-fixed and routinely processed for hematoxylin-eosin (HE) staining and immunohistochemistry (IHC) analysis. The Gleason score (ISUP grade) was considered as the highest score on the biopsy specimen. The histopathological results served as a reference and were stratified in accordance with the 7^th^ edition of the American Joint Committee on Cancer (AJCC) staging system for PC (23). The pathological results were confirmed by the consensus of two board-certified specialists in genitourinary pathology, as previously reported (24). The pathologists were blinded to both the ^68^Ga-PSMA PET/CT results and the clinical evaluation of the tissues from the surgeons.

### Immunohistochemistry Staining

The tissue samples were formalin-fixed and routinely processed for IHC staining to evaluate PSMA expression with anti-PSMA antibody (1:100, MAB-0575, MXB Biotechnologies), as previously reported (25). Further methods related to IHC are included in the **Supplementary Materials** and *Methods*.

### Immunofluorescence Staining

The slides were processed for IF staining of PSMA (1:50, MAB-0575, MXB Biotechnologies) and p504s (a biomarker of PC, 1:50, RMA-0546, MXB Biotechnologies), a technique that was previously reported (26). Details regarding IF procedures are included in the **Supplementary Materials** and *Methods*.

### Imaging Evaluation of mpMRI and Bone Scan

All mpMRI evaluations were performed on a 3.0-T MR scanner (Achieva 3.0 T TX, Philips Medical Systems, The Netherlands) by using a 16-channel phased-array coil, as we previously described (27). LNs were rated as malignant if they have a short-axis diameter > 10 mm and if they showed restricted diffusion on the DWI and ADC map or increased contrast enhancement (28). Bone scan was performed on Symbia T2 (Siemens Medical Solutions, Erlangen, Germany) with at 3-5 hours after injection of 20-25mCi ^99^Tc^m^-MDP. The evaluation of mpMRI or bone scan were reviewed by two radiologists or two board-certified nuclear medicine specialists

### Imaging Protocol and Evaluation of ^68^Ga-PSMA PET/CT

All ^68^Ga-PSMA PET/CT evaluations were performed at a single center (Fourth Military Medical University, Xijing Hospital, Xi’an, Shaanxi, China). Patients underwent ^68^Ga-PSMA PET imaging on a Biograph 40 system (Siemens Medical Solutions, Erlangen, Germany). The ^68^Ga/^68^Ge generator system was produced by ITG GmbH (Munich, Germany), and the DOTA ligand was acquired from ABX GmbH (Radeberg, Germany). The ^68^Ga-PSMA-617 was synthesized as we previously reported (24), and the patients were intravenously injected with 1.8-2.2 MBq/kg body weight ^68^Ga-PSMA-617. Mean injection activity of ^68^Ga-PSMA PET was 141.7 ± 21.9 MBq. Low-dose CT (pitch 0.8, 50 mA, 120KV[peak]) scans for PET attenuation were obtained (automatic mA, 120keV, 512x512 matrix, 5-mm slice thickness, 1.0-s rotation time, and 0.8 pitch), followed by a PET scan with 5 bed positions (3 min/bed, from head to the proximal thighs) performed about 60 minutes after tracer injection. The PET/CT images were then transferred to a multimodal workstation for data analysis (Syngo Truepoint Siemens Medical Solutions).

The scans of ^68^Ga-PSMA PET/CT were reviewed by two board-certified nuclear medicine specialists (Z.Q. and F.K.) with more than ten years’ experience in reading PET imaging and one board-certified radiation oncologist (J.W.). According to prior studies, lymph nodes with a SUV_max_ of 2.0 or more and a diameter of 5 mm or more were considered PSMA-positive on ^68^Ga-PSMA PET (29, 30). Scans were evaluated using a Siemens MIWP workstation (Syngo MIWP; Siemens Medical Solutions, Erlangen, Germany), according to the Joint EANM and SNMMI procedure guidelines (version 1.0) (31, 32).

### Statistical Analysis

Descriptive statistics were calculated and presented as the frequency (percentage) for categorical variables, the mean (standard deviation) for continuous variables of normal distribution and the median (quartile) for continuous variables of skewness distribution. All data were analyzed by IBM SPSS statistics software, version 23.0 (IBM, Inc., Chicago, IL, USA).

## Results

### Patient Characteristics and Pattern of Metastatic Spread

**Figure 1:**
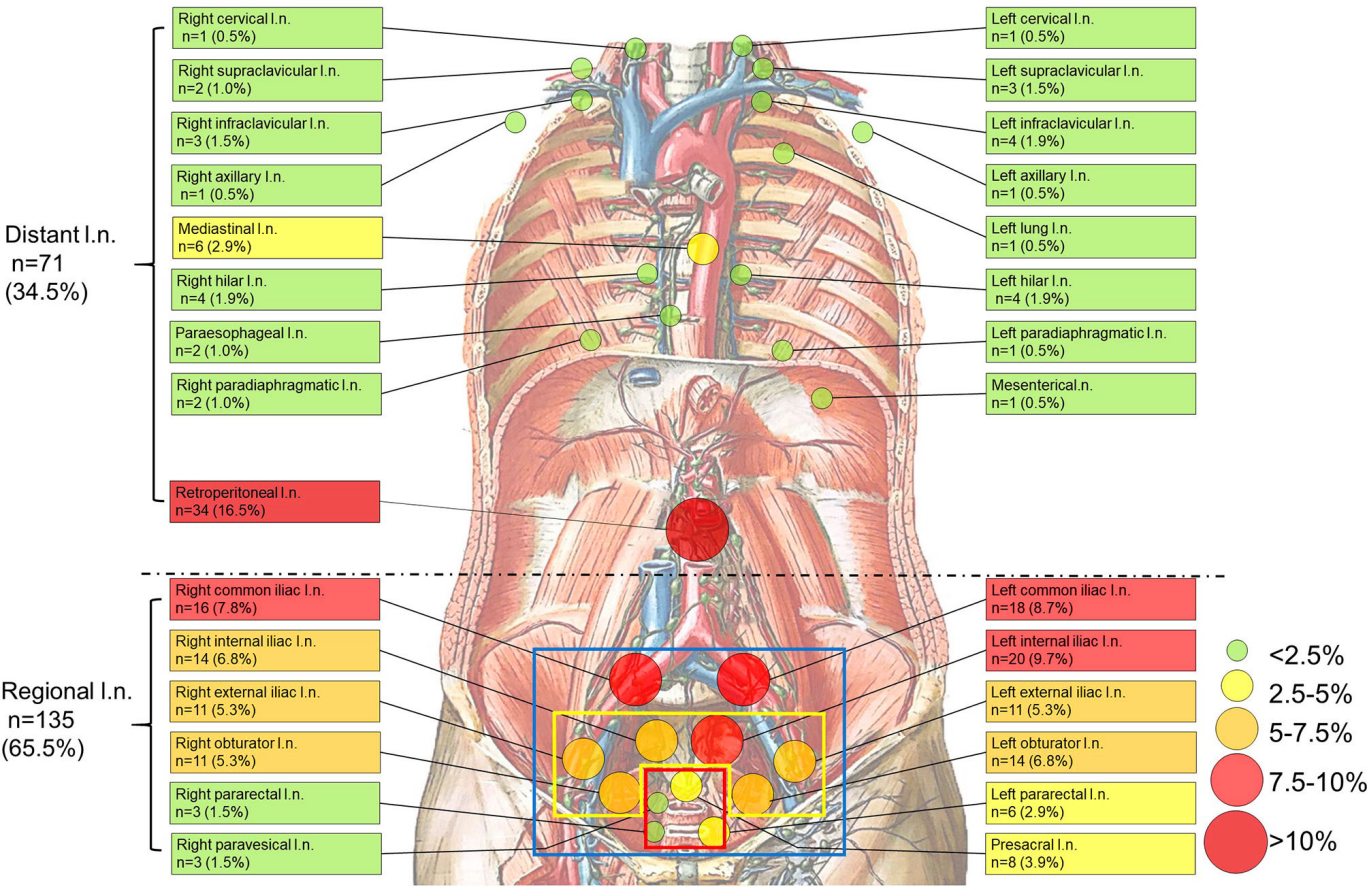
Anatomical distribution of PSMA-avid lymph node metastases in the whole body. (blue square, the scope of ePLND; yellow square, the scope of limited PLND; red square, the selective resected lymph nodes if radioactive in sentinel PLND). Anatomical distribution of non-regional PSMA-avid lymph node metastases. All non-regional lymph node metastases (n=71, 34.5%) are beyond pelvic mpMRI, the currently recommended imaging by the guidelines (7, 9). Background picture (Lymph Vessels and Nodes of Posterior Abdominal Wall) was used under the permission of ^©^ Elsevier Inc.

**Figure 2:**
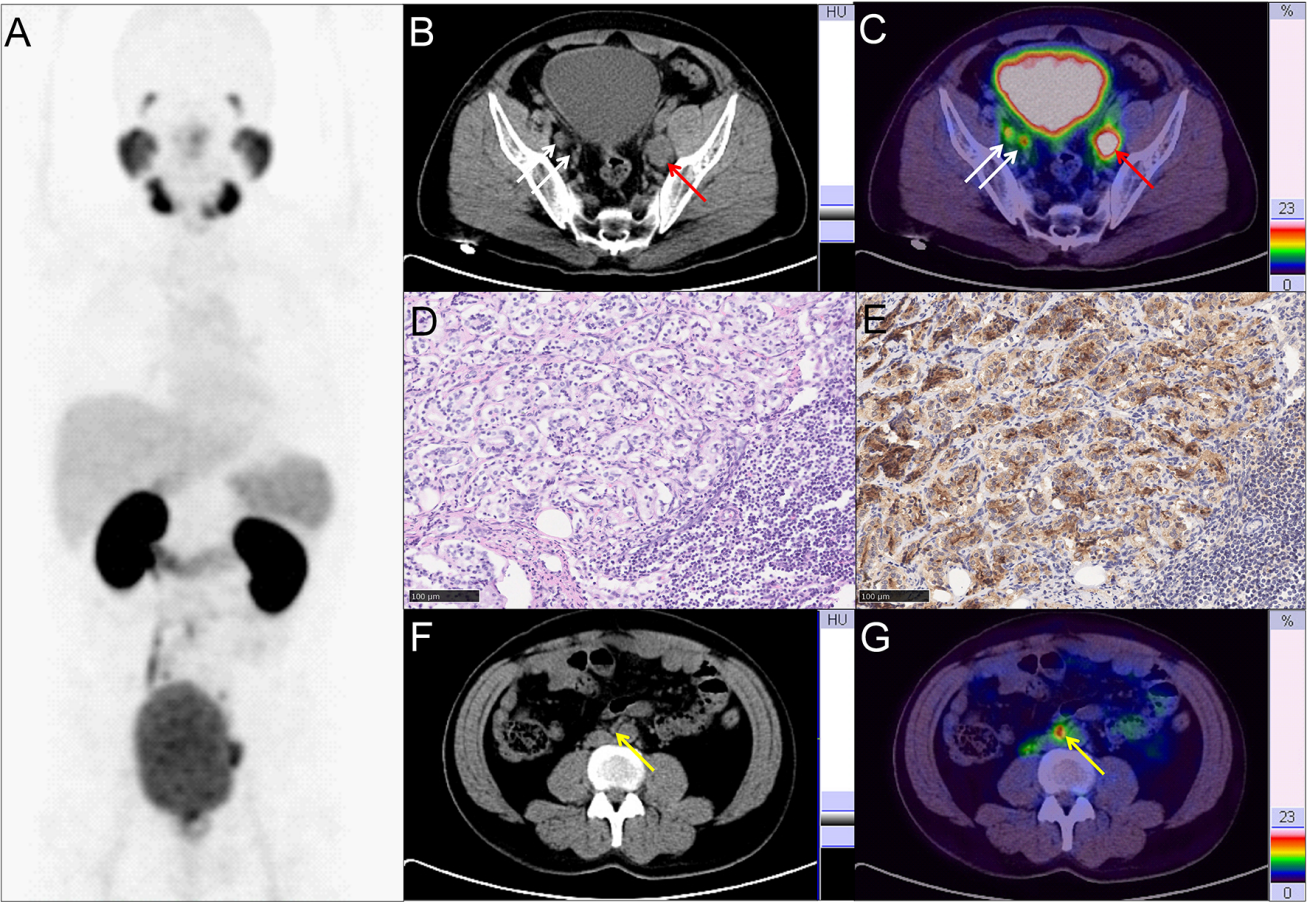
Representative PC patient with non-regional oligometastatic lymph nodes. **(A–C, F, G)** ^68^Ga-PSMA PET/CT results of a 40 y/o PC patient with LNMs (cT3a, GS 4 + 4 = 8, tPSA 47.99 ng/ml). **(B, C)** Typical para-iliac LNMs (white arrow, right para-iliac LNMs; red arrow, typical left para-iliac LNMs, SUV_max_ 13.2). **(D)** HE staining and **(E)** PSMA staining of the resected left para-iliac LNMs. **(F, G)** non-regional oligometastatic LNMs in paraaortic area (yellow arrow, SUV_max_ 10.9). The LNMs risks were 94.0%, 93.0%, and 89.0% according to the Briganti, MSKCC and Winter nomograms, respectively. The LNMs risk (>65%) indicated that the PC patients may have non-regional LNMs. After ^68^Ga-PSMA PET/CT, radiotherapy was also performed on the oligometastatic paraaortic LNM after RP with ePLND.

The characteristics of the 57 patients included in the study are summarized in **Table 1**. To better understand the pattern of metastatic spread of LNMs, we depicted non-regional LNMs from all PC patients (**Figure 1**). ^68^Ga-PSMA PET/CT visual analysis found 206 PSMA-positive LNMs from 49.1% (28/57) of the patients (mean 7.4 nodes per patient; range: 1-30 positive nodes per patient). A representative PC patient with non-regional oligometastatic lymph nodes is shown in **Figure 2** and a PC patient without LNMs is shown in **Figure S1**. **Figure S2** shows a representative PC patient with LNMs had negative results in mpMRI but positive results in ^68^Ga-PSMA PET/CT. If staining confirmed co-expression of PSMA and P504s, a biomarker of PC cells, on resected LNMs (**Figure S4**).

**Table 1:** Characteristics of patients and tumors at diagnosis.

Characteristic		value
Age (ages)		
	Mean ± SD	68.5 ± 8.2
	Median (range)	69.0 (40-84)
tPSA at PSMA PET/CT (ng/mL)		
	Median (P_25_-P_75_)	30.7 (8.8-149.5)
SUV_max_		
	Mean ± SD	21.3 ± 19.2
	Median (P_25_-P_75_)	16.0 (7.9-25.1)
Injection dose (MBq)		
	Mean ± SD	141.9 ± 21.5
	Median (range)	142.1(67.0-181.3)
Uptake time (minutes)		
	Mean ± SD	66.617 ± 14.5
	Median (range)	62(40-98)
T-stage, n (%)		
	T2a	5 (8.8%)
	T2b	5 (8.8%)
	T2c	38 (66.7%)
	T3a	2 (3.5%)
	T3b	4 (7.0%)
	T4	3 (5.3%)
Gleason score, n (%)		
	6	3 (5.3%)
	3+4 = 7	2 (3.5%)
	4+3 = 7	8 (14.0%)
	8	22 (38.6%)
	9	17(29.8%)
	10	5(8.8%)
Risk-group according to D’Amico, n (%)		
	High	57(100.0%)

### The Detection and Distribution of Non-Regional LNMs by ^68^Ga-PSMA PET/CT

To clarify the non-regional LNMs detected by ^68^Ga-PSMA PET/CT and its distribution, we depicted non-regional LNMs from all PC patients (**Figure 1**). For 57 patients, 34.5% (71/206) of the LNMs were non-regional and 65.5% (135/206) of the LNMs were regional.

**Figure 3:**
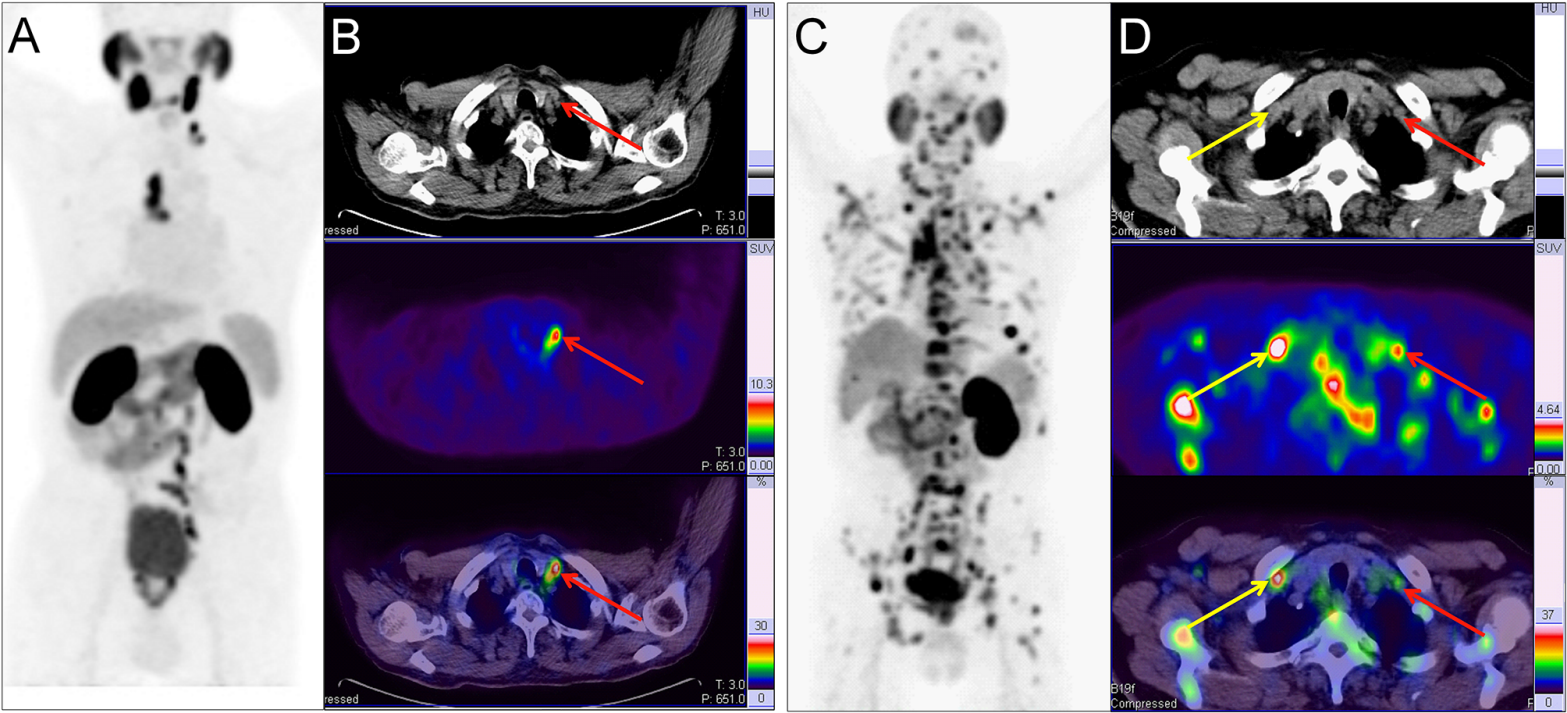
Two example PC patients with Virchow nodes. **(A, B)** ^68^Ga-PSMA PET/CT results of a 76 y/o patient (T2cN1M1a, GS 4 + 4 = 8, tPSA 150.9 ng/ml). Virchow nodes (red arrow, SUV_max_ 8.42). After ^68^Ga-PSMA PET/CT, LNMs were shown in the Virchow nodes, mediastinal lymph nodes, and retroperitoneal lymph nodes so RP with ePLND could be replaced by RT. **(C, D)** ^68^Ga-PSMA PET/CT of a 73 y/o patient (pT4N1M1b; GS 5 + 4 = 9; tPSA 747.9 ng/ml). right supraclavicular LNMs (yellow arrow, SUV_max_ 7.89) and Virchow nodes (red arrow, SUV_max_ 2.98). In the first patient, the LNMs risks were 81.0%, 99.0%, and 70.0% according to the Briganti, MSKCC and Winter nomograms, respectively. The LNMs risk (>65%) indicated that the PC patients may have non-regional LNMs. After ^68^Ga-PSMA PET/CT, the radiation scope of radiotherapy could be modified for more accurate location.

As shown in **Table 2**, non-regional LNMs were observed in 57.1% (16/28) of the PC patients with LNMs. Nearly half of all LNMs occurred in retroperitoneal lymph nodes (n=34, 16.5%), while the remaining LNMs (n=37, 18.0%) were observed in other areas above the diaphragm. The most commonly observed LNMs above the diaphragm were seen in the mediastinal area (n=6, 2.9%). PSMA-avid LNMs in the left infraclavicular lymph nodes, the left hilar lymph nodes, and the right hilar lymph nodes were equally observed (n=4, 1.9%). PSMA-positive LNMs were also observed in the left supraclavicular lymph nodes (Virchow nodes, n=3, 4.2%) and the right infraclavicular lymph nodes (n=3, 4.2%). A patient with Virchow nodes is shown in **Figures 3A, B**, and a patient with bilateral supraclavicular LNMs is shown in **Figures 3C, D**. In addition, LNMs were observed in the left/right cervical lymph nodes, the right supraclavicular lymph nodes, the left/right axillary lymph nodes, the left/right hilar lymph nodes, the paraesophageal lymph nodes, and the left/right paradiaphragmatic lymph nodes. One mesenteric lymph node and one metastatic node of the left lung were also observed. In total, more than one-third of all LNMs were non-regional LNMs, which were beyond the range of PLND (**Figure 1**, **Table 2**).

**Table 2:** Overview of distant lymph nodes metastases.

Location	∑
Total	71 (100.0%)
left supraclavicular lymph nodes	3(4.2%)
right supraclavicular lymph nodes	2(2.8%)
left infraclavicular lymph nodes	4(5.6%)
right infraclavicular lymph nodes	3(4.2%)
left cervical lymph nodes	1(1.4%)
right cervical lymph nodes	1(1.4%)
paraesophageal lymph nodes	2(2.8%)
left axillary lymph nodes	1(1.4%)
right axillary lymph nodes	1(1.4%)
left hilar lymph nodes	4(5.6%)
right hilar lymph nodes	4(5.6%)
left lung	1(1.4%)
mediastinal lymph nodes	6(8.5%)
left paradiaphragmatic lymph nodes	1(1.4%)
right paradiaphragmatic lymph nodes	2(2.8%)
mesenteric lymph nodes	1(1.4%)
Retroperitoneal lymph nodes	34(47.9%)

Thus, more than one-third of all LNMs shown in the ^68^Ga-PSMA PET/CT were non-regional LNMs, which were beyond the range of PLND. Totally, 28.1% (16/57) of all patients have both regional and non-regional LNMs, and no skip metastases was observed in each patient.

### The Evaluation of Regional LNMs Risks According to Three Clinical Nomograms

To compare the scores of having regional LNMs, we calculated the risks of having LNMs for the patients in the study according to the three PLND-based nomograms (5, 6, 33).

**Figure 4:**
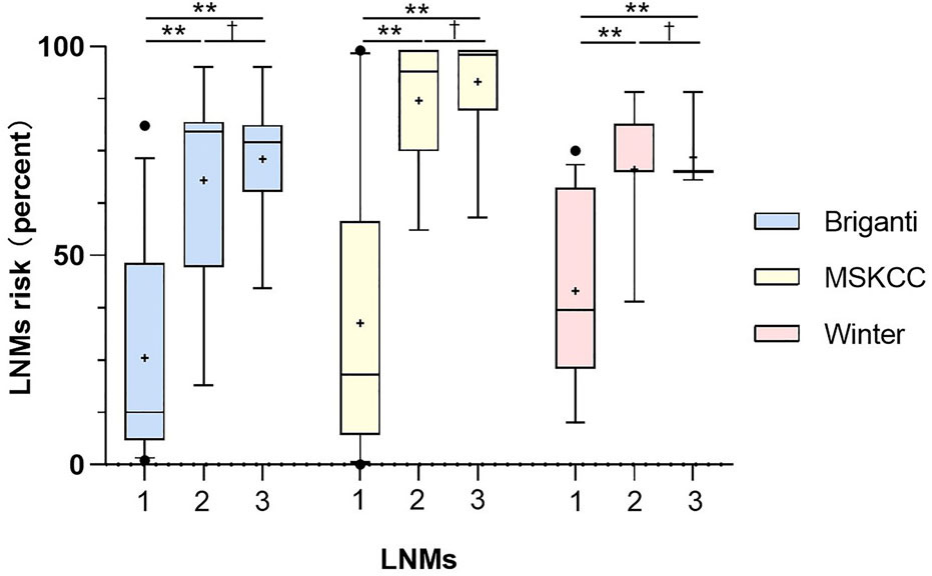
Comparison of risks from the three nomograms between the patients with no LNMs (1), the patients with regional LNMs (2) and the patients with distant LNMs (3). Vertical borders of box represent 25th and 75th percentiles and middle bar represents median while “+” represents mean. Mann-Whitney U tests showed significant difference between the patients with LNMs and without LNMs (**P<0.001) and the PC patients with non-regional LNMs have higher scores than those with regional LNMs. The LNMs risks were calculated from Briganti, MSKCC and Winter nomograms.

As shown in **Figure 4**, the PC patients with nonregional LNMs have higher scores than those without LNMs. The risks of LNMs according to the Briganti, MSKCC and Winter nomograms were 48.0% (median; range 1.0-95.0%), 63.0% (median; range 9.0-99.0%), and 70.0% (median; range 10.0-89.0%), respectively (**Table 3**). For the Briganti nomogram, the risks varied from low (<10.0%) for 13 men (22.8%) to very high (>50.0%) for 25 men (43.9%) in our cohort. For the MSKCC nomogram, the risks varied from low (<10.0%) for 10 men (17.5%) to very high (>50.0%) for 35 men (61.4%) in our cohort. For the Winter nomogram, the risks varied from low (<10.0%) for 2 men (3.5%) to very high (>50.0%) for 35 men (61.4%). Due to high tPSA values or GS, the risk of LNMs in 18 patients was more than 90.0% from the MSKCC nomogram.

**Table 3:** Risk of lymph nodes metastases according to three clinical nomograms.

		Briganti nomogram	MSKCC nomogram	Winter nomogram
Patient number		57	57	57
Median (range) [%]		48.0 (1.0-95.0)	63.0 (0.0-99.0)	70.0 (10.0-89.0)
Subgroups				
≤10.0%		13(22.8%)	10(17.5%)	2(3.5%)
10.1-20.0%		7(12.3%)	4(7.0%)	1(1.8%)
20.1-30.0%		3(5.3%)	5(8.8%)	10(17.5%)
30.1-40.0%		1(1.8%)	1(1.8%)	8(14.0%)
40.1-50.0%		8(14.0%)	2(3.5%)	1(1.8%)
50.1-60.0%		4(7.0%)	6(10.5%)	1(1.8%)
60.1-70.0%		6(10.5%)	4(7.0%)	30(52.6%)
70.1-80.0%		3(5.3%)	2(3.5%)	1(1.8%)
80.1-90.0%		10(17.5%)	5(8.8%)	3(5.3%)
>90.0%		2(3.5%)	18(31.6%)	0 (0.0%)
Unknown		0 (0.0%)	0 (0.0%)	0 (0.0%)

Thus, clinical nomograms indicated that most of the patients in this study need to receive ePLND to deal with regional LNMs or potential regional LNMs. The PC patients with distant LNMs has higher scores than those with regional LNMs. Then, we tried to clarify the non-regional LNMs by ^68^Ga-PSMA PET/CT.

### The Tangible Benefit of ^68^Ga-PSMA PET/CT in Nomogram-Based Therapy Choices of High-Risk Prostate Cancer Patients With Non-Regional Lymph Node Metastases

Next, we analyzed whether an additional ^68^Ga-PSMA PET/CT have potential benefit on nomogram-based therapy choices of high-risk PC patients, including sparing unnecessary ePLND in PC patients with non-regional LNMs. Four examples were used to show the potential benefit on clinical decision-making, especially in those with non-regional LNMs.

Therapy modality changes were made in the PC patients with non-regional oligometastatic lymph nodes. As shown in **Figure 2**, a 40-year-old patient (T3a stage) with tPSA 48.0 ng/ml and GS 4 + 4 = 8 with pelvic LNMs on pelvic mpMRI and no BMs on bone scan. The LNMs risks were 94.0%, 93.0%, and 89.0% according to the Briganti, MSKCC and Winter nomograms, respectively. As a result, the patient underwent RP and extended PLND (ePLND) according to standard guidelines (7, 9). However, ^68^Ga-PSMA PET/CT revealed that non-regional oligometastatic lymph nodes existed in the paraaortic area. Therefore, after surgery, radiotherapy (RT) should be performed on the paraaortic LNMs.

As another example, changes in therapeutic modality may also occur in PC patients based on the detection of non-regional LNMs. As shown in **Figure 3A**, a 76-year-old patient (T2c stage) with tPSA 150.9 ng/ml and GS 4 + 4 = 8 with regional LNMs on pelvic mpMRI and no BMs on bone scan. The LNMs risks were 81.0%, 99.0%, and 70.0% according to the Briganti, MSKCC and Winter nomograms, respectively. As a result, the patient received RP and ePLND according to standard guidelines (7, 9). However, ^68^Ga-PSMA PET/CT revealed non-regional LNMs in the Virchow nodes, mediastinal lymph nodes, and retroperitoneal lymph nodes. Hence, RP and ePLND should be replaced by image-guided radiotherapy (IGRT). ^68^Ga-PSMA PET/CT has the potential to reduce unnecessary ePLND in this kind of PC patients.

In addition, changes in RT scope may be recommended for PC patients with many non-regional LNMs. As shown in **Figure 3B**, a 73-year-old patient (T4 stage) with tPSA 747.9 ng/ml and GS 4 + 5 = 9 with regional LNMs on pelvic mpMRI and BMs on bone scan. However, all non-regional LNMs, such as Virchow nodes, were missed by pelvic mpMRI. With ^68^Ga-PSMA PET/CT, non-regional LNMs were clearly shown for accurate modification of the radiation area of IGRT.

Furthermore, ^68^Ga-PSMA PET/CT has the potential to reduce unnecessary ePLND in PC patients without LNMs. As shown in **Figure S1**, a 71-year-old patient (T2a stage) with tPSA of 9.3ng/ml and GS 4 + 4 = 8 with no pelvic LNMs on pelvic mpMRI and no BMs on bone scan. The LNMs risks were 11.0%, 21.0%, and 39.0% according to the Briganti, MSKCC and Winter nomograms, respectively. Therefore, the patient underwent RP and ePLND to treat the potential LNMs according to the guidelines (7, 9). However, no LNMs were observed on ^68^Ga-PSMA PET/CT. After ePLND, the pathological results from resected lymph nodes confirmed that no LNMs existed, which were in agreement with the PSMA PET/CT findings.

**Figure 5:**
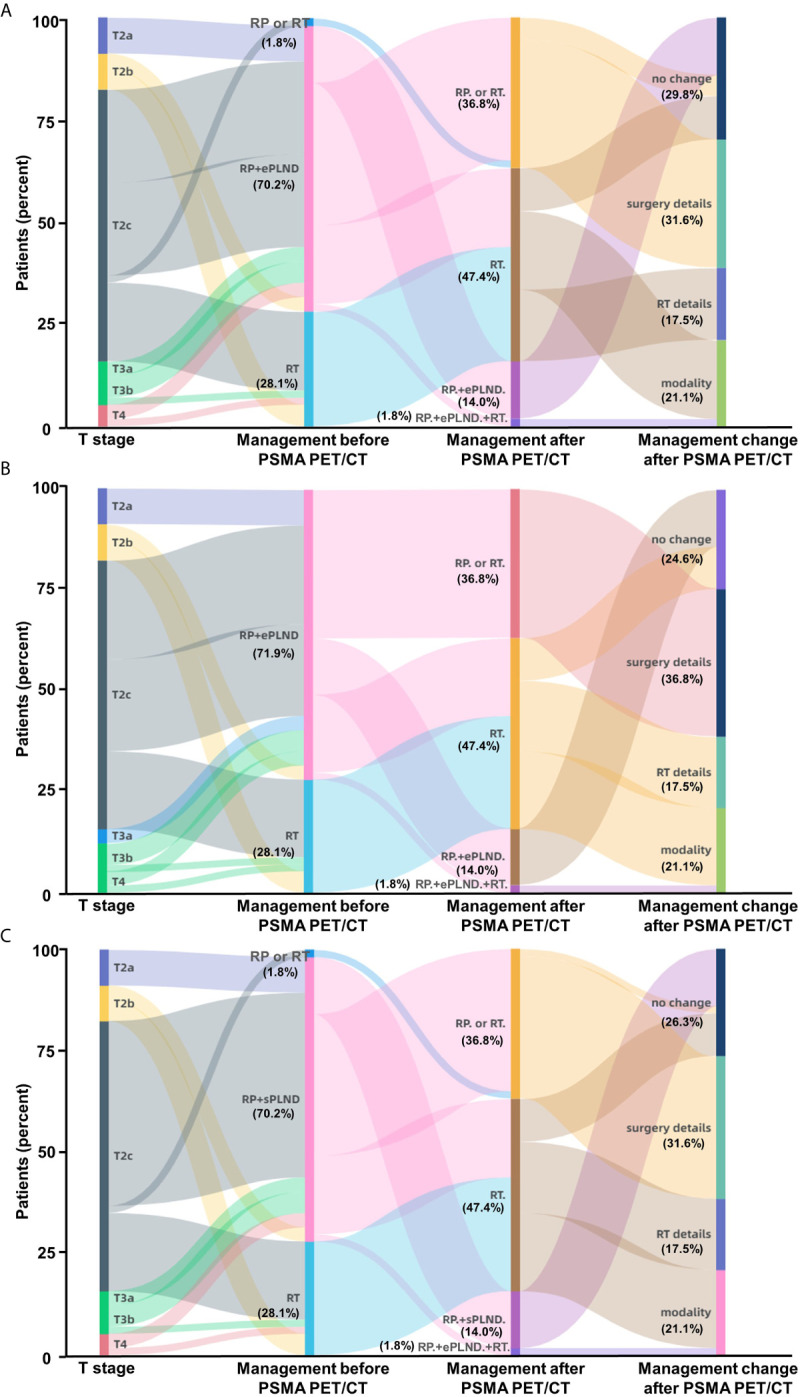
Impact of ^68^Ga-PSMA PET/CT on clinical decision-making in high-risk treatment-naïve prostate cancer patients. **(A)** According to the Briganti nomogram, ^68^Ga-PSMA PET/CT would have led changes of planned therapy in 70.2% of these patients and avoided unnecessary ePLND in 12.3% of the patients who have previously undetected non-regional LNMs. **(B)** According to the MSKCC nomogram, ^68^Ga-PSMA PET/CT would have led changes of planned therapy in 75.4% of the patients; **(C)** According to the Winter nomogram, ^68^Ga-PSMA PET/CT would have led changes of planned therapy in 73.7% of the patients. As the basic treatment for nearly all high-risk PC patients, ADT or systemic therapy was not included. (Modality: changes of therapy modality; surgery details: changes in surgery details; RT details: change in RT field; RP: radical prostatectomy; RT: radiotherapy; ePLND: extend PLND; sPLND: sentinel PLND).

In general, the new information of ^68^Ga-PSMA PET/CT can lead to changes of therapy choices in 70.2% (40/57) of the PC patients when using the Briganti nomogram in clinical decision-making (**Figure 5A**). Therapy modality changes could be made in 21.1% of the patients and other changes include details of RT scope (17.5%) or type and extent of surgery (31.6%). Changes in therapeutic treatment modality were due to newly detected non-regional LNMs in 12.3% of the patients and newly detected BMs in 8.7% of the patients. ^68^Ga-PSMA PET/CT has the potential to reduce unnecessary ePLND in 12.3% of the patients because of newly detected non-regional LNMs. Newly detected non-regional LNMs can also lead to modification of RT in 17.5% of the patients. Similarly, 75.4% (43/57) of the patients would have had different clinical decision-making as compared to the MSKCC nomogram (**Figure 5B**) and 73.7% (42/57) of the patients would have had different clinical decision-making as compared to the Winter nomogram (**Figure 5C**) after ^68^Ga-PSMA PET/CT. Therefore, ^68^Ga-PSMA PET/CT can provide added benefit to the three standard nomograms and affect clinical decision-making, especially for patients with non-regional LNMs.

### The Establishment of New Thresholds for PLND-Validated Clinical Nomograms to Predict Non-Regional Lymph Node Metastases

According to the guidelines, the PC patients with LNMs risk higher than 5% in Briganti nomogram, 2% in MSKCC nomogram or 7% in Winter nomogram need receive ePLND to deal with potential regional LNMs (5–7). However, whether the PLND-validated clinical nomograms have the potential to evaluate the risk of non-regional LNMs remains unclear. The PC patients with LNMs risk higher than the above mentioned cutoff values, such as 5%, may also have non-regional LNMs and these patients with non-regional LNMs should not receive ePLND. Next, we analyzed whether the clinical nomograms have the potential to predict non-regional LNMs.

**Figure 6:**
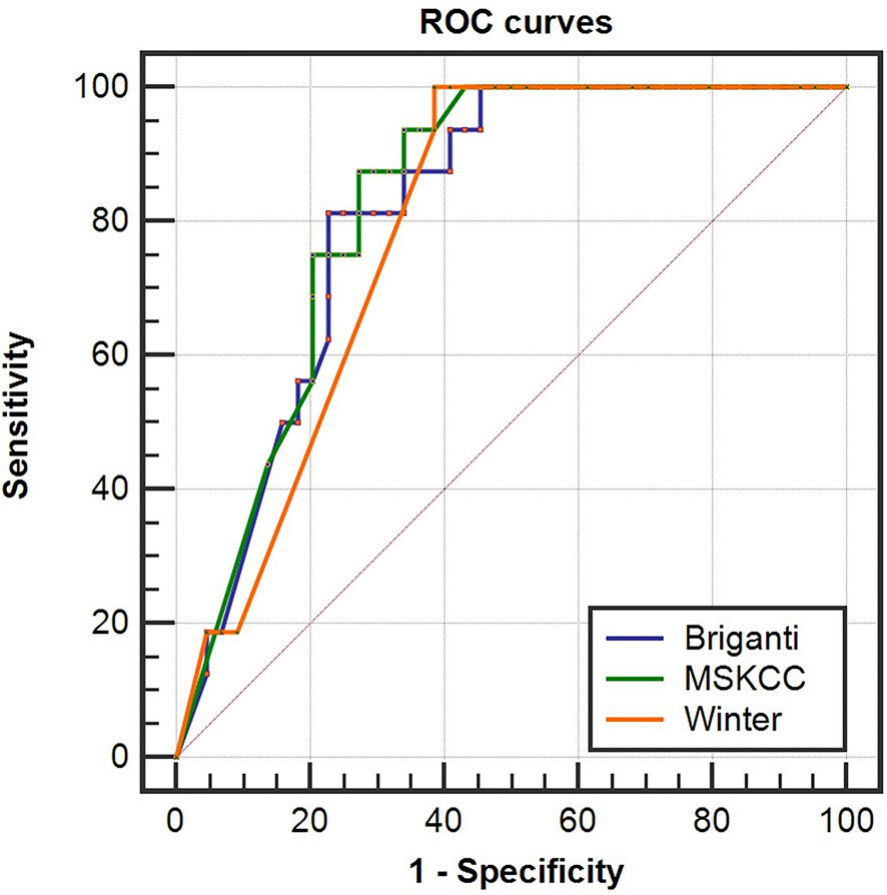
Receiver operating characteristic (ROC) curves of the Briganti, MSKCC and Winter nomograms for predicting non-regional PSMA PET positive LNMs in high-risk PC patients. The AUCs of the clinical nomograms (Briganti, MSKCC and Winter) in predicting non-regional LNMs were 0.816, 0.830 and 0.793, respectively. The PC patients with a score >64% in Briganti nomogram, a score >75% in MSKCC nomogram and a score >67% in Winter nomogram were more likely to have non-regional LNMs. The above cutoff values can be used to predict non-regional LNMs in high-risk PC patients.

**Figure 7:**
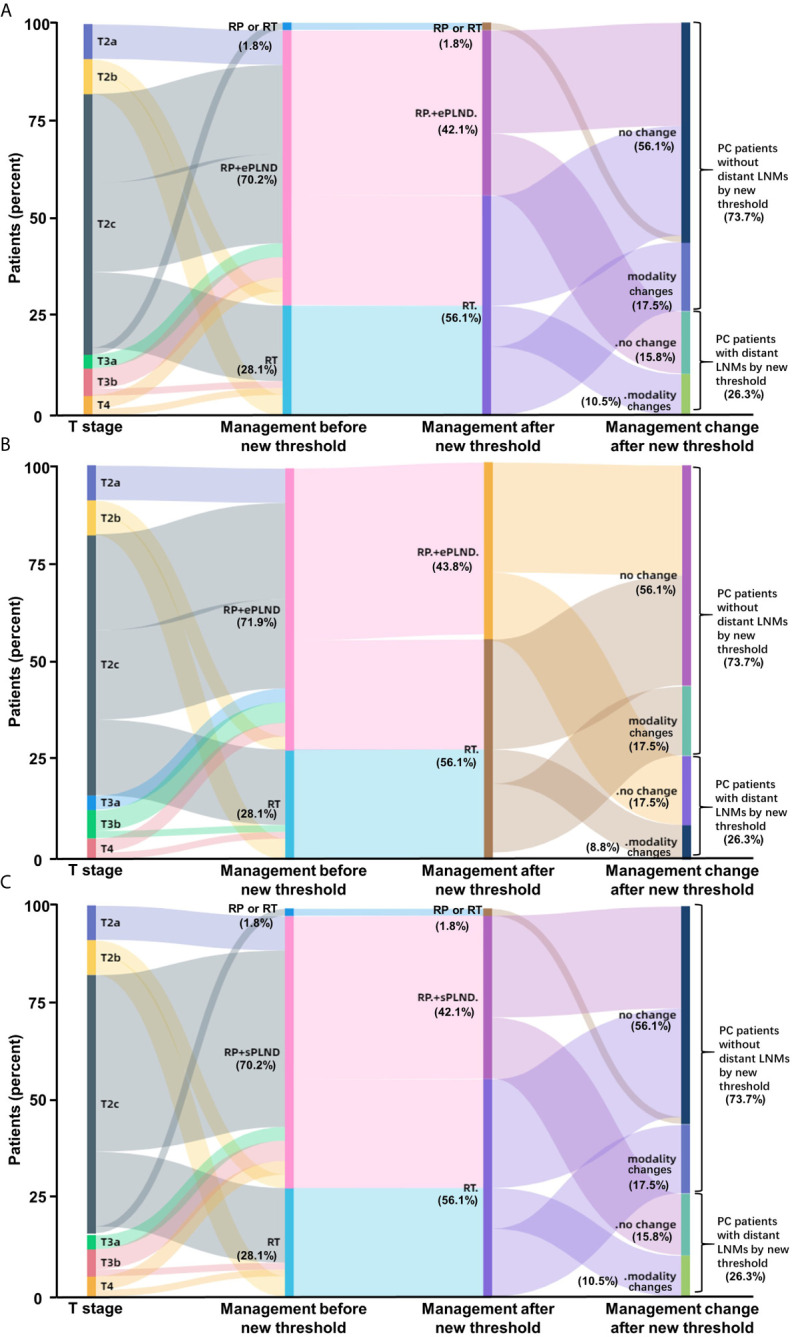
Impact of new thresholds for PLND-validated clinical nomograms on clinical decision-making in high-risk treatment-naïve prostate cancer patients (**A**: Briganti nomogram; **B**: MSKCC nomogram; **C**: Winter nomogram). The new thresholds in Briganti **(A)** and Winter **(C)** nomograms excluded 93.75% (15/16) of the PC patients with non-regional LNMs and 87.5% (14/16) of the patients for MSKCC **(B)** nomogram. (Modality, changes of therapy modality; RP, radical prostatectomy; RT, radiotherapy; ePLND, extend PLND; sPLND, sentinel PLND).

To determine whether the clinical scores of nomograms can predict PSMA PET positive non-regional LNMs by setting a higher cutoff value, we plotted the ROC curves of the three nomograms to compare the accuracy of nomograms in predicting PSMA PET positive non-regional LNMs; for each nomogram, a cutoff value corresponding to highest level accuracy was utilized. The AUC of the clinical scores to predict PSMA PET positive non-regional LNMs is shown in **Figure 6**. We found that the PC patients with a score >64% in Briganti nomogram, a score >75% in MSKCC nomogram and a score >67% in Winter nomogram were more likely to have non-regional LNMs. We found the AUC of MSKCC and Briganti nomograms was slightly higher than those of Winter nomogram. The AUC of clinical nomograms was shown in **Table 4**. Using the above higher cutoff values, 10.5% (6/57) high-risk PC patients with non-regional LNMs can be excluded by Briganti and Winter nomograms, and 8.7% (5/57) high-risk PC patients with non-regional LNMs can be excluded by MSKCC nomograms (**Figure 7**).

**Table 4:** Distant PSMA PET positive LNMs of ROC analyses by three clinical nomograms.

Stage (number)	Characteristics	AUC	SE	95% CI
T1-T3 (n=57)	MSKCC nomogram	0.830	0.051	0.710-0.914
	Briganti nomogram	0.816	0.054	0.695-0.904
	Winter nomogram	0.793	0.05	0.668-0.886

Conclusively, the ePLND-based clinical nomograms have the potential to predict non-regional LNMs as well. The surgeons should pay close attention to the PC patients with LNMs risk higher than approximately 65% for potential non-regional LNMs.

## Discussion

This study depicted the pattern of metastatic spread by ^68^Ga-PSMA PET/CT and demonstrated the potential benefit of this imaging modality on clinical decision-making for high-risk PC patients, especially in patients with non-regional LNMs. ^68^Ga-PSMA PET/CT can provide pattern of metastatic spread of LNMs with a higher sensitivity and specificity compared with conventional morphological imaging (34). One previous study indicated that, compared with mpMRI, more LNMs can be observed by ^68^Ga-PSMA PET/CT in 27.8% of PC patients (35). In this study, 96.7% of the patients with LNMs who received ^68^Ga-PSMA PET/CT had detectable LNMs, while pelvic mpMRI could only detect regional LNMs in 60.7% of patients. Therefore, pelvic mpMRI alone has limited sensitivity for detection of regional LNMs and cannot include non-regional LNMs.

In our study, 34.5% of the LNMs were detected in non-regional sites. This finding was comparable to prior work, including a study of 280 treatment-naïve PC patients that reported the use of ^68^Ga-PSMA PET/CT detected 36.0% of LNMs as non-regional and 15.5% of LNMs above the diaphragm (8). The most commonly observed non-regional LNMs were the paraaortic lymph nodes (12.8%), while the most commonly encountered non-regional LNMs above the diaphragm were the mediastinal lymph nodes (6.2%) (8). Our results were consistent with the prior study because 97.8% of the patients in this work were intermediate- to high-risk PC patients (8). In another study, ^68^Ga-PSMA PET/CT revealed that 16.0% of the treatment-naïve PC patients had LNMs and 6.0% of these patients had non-regional LNMs (36). Meanwhile, Nurhan et al. reported that 9.0% of the treatment-naïve PC patients had non-regional LNMs (37). For the intermediate- to high-risk treatment-naïve PC patients, one study indicated that 23.3% of the patients had regional LNMs and 9.5% of them had non-regional LNMs (38). One previous study showed that retroperitoneal LNMs were encountered in 12.8% of the high-risk PC patients by ^68^Ga-PSMA I&T PET/CT (39). In our study, the retroperitoneal LNMs were the most commonly visualized non-regional LNMs. We identified 57.1% compared to 47.7% (in BJU study) non-regional LNMs of the PC patients with LNMs (40). Compared with previous studies, more non-regional LNMs were observed in our study because more high-risk PC patients with higher grade of PC were included in our cohort. Furthermore, we focused on that a threshold can be set for current clinical PLND-validated nomograms to predict extrareolar LN metastases with an AUC accuracy of about 80% after optimizing the simple nomograms which may help to improve the efficiency for PC therapy significantly in clinical practice. PSMA expression, which could be reflected by Maximum Standardized Uptake Values (SUV_max_) of the primary tumor in ^68^Ga-PSMA PET/CT, was closely correlated with higher grades of PC (41). As shown in **Figure S3**, a significantly higher SUV_max_ was found in the patients with LNMs versus those without LNMs (*P*<0.01, Mann-Whitney-U-test). The Virchow nodes were observed in our study (**Figure 3**). One previous study also reported that 3.2% of the treatment-naïve patients have LNMs in the Virchow nodes (8). The study showed that the Virchow nodes were significantly more frequent with a GS ≥8 as compared to a GS ≤7b primary PC tumor (8, 42). In our study, all PC patients with Virchow nodes had a GS ≥8.

The new information obtained *via* ^68^Ga-PSMA PET/CT can benefit clinical decision-making and provide additive benefit to existing PLND-validated nomograms, particularly for individuals with non-regional LNMs. For treatment-naïve PC patients, the most frequent new findings were LNMs (17.2%) and ^68^Ga-PSMA PET/CT can impact therapeutic decision making in 27.6% of patients (38). In our study, based on the ^68^Ga-PSMA PET/CT findings, the new information could have resulted in a change in clinical management in more than two-thirds of patients. In our study, the newly detected non-regional LNMs by ^68^Ga-PSMA PET/CT can lead to therapy modality change in 12.3% of the patients and modification of RT in 17.5% of the patients. In previous work, treatment-naïve PC patients and recurrent PC patients were not studied separately and most prior studies focused on RT management exclusively. Florian et al. reported 27.5% of the PC patients had non-regional LNMs, and the new information from ^68^Ga-PSMA PET/CT led to radiotherapeutic management in 50.8% of the cases (43). In this study including 26.0% treatment-naïve patients, the new information of ^68^Ga-PSMA PET/CT led to changes of planned RT in 26.4% of the patients (43). In another study including 48.1% treatment-naïve patients, ^68^Ga-PSMA PET/CT changed RT in 46.3% of the cases, and changed hormone therapy in 33.3% of the patients, with an overall change in decision-making in 53.7% of the patients (44). In an Australian prospective multi-center study, ^68^Ga-PSMA PET/CT led to changes of management intent in 21.3% of the treatment-naïve patients while 61.5% of the patients with biochemical recurrence had changes in planned therapy (45). More metastatic lesions were discovered in high-risk PC patients to facilitate RT planning by mapping PSMA-avid lesions (46). One study indicated that ^68^Ga-PSMA PET/CT changed TNM stage and RT in 26.0% and 44.0% of the 50 treatment-naïve PC patients, respectively (36). In our study, ^68^Ga-PSMA PET/CT also revealed the potential to reduce ePLND in 31.6% of the patients. Similarly, one previous study demonstrated that ^68^Ga-PSMA PET imaging had the potential to facilitate patient selection for ePLND in intermediate- to high-risk PC patients, and these authors created a novel PLND-validated nomogram including tPSA, GS, and PSMA positive volume (PSMA_total_) of ^68^Ga-PSMA PET/CT (47). The therapy modality changes and therapy detail changes were modified from one previous study (38).

In the current study, our study demonstrated that the PLND-validated clinical nomograms (Briganti, MSKCC, Winter) have the potential to predict non-regional LNMs with a higher cutoff value of scores, although the three clinical nomograms are widely used to predict the risks of regional LNMs in previous studies (5–7). The PC patients with LNMs risk higher than 65% need to receive PSMA PET/CT to exclude non-regional LNMs to reduce unnecessary ePLND.

Our study had some limitations. The first limitation in this study is that ^68^Ga-PSMA PET/CT may have limitation on detecting LNMs in the prostate cancer patients with negative PSMA expression (like neuroendocrine prostate cancer), although expression of PSMA has been recognized in approximately 95% of prostate cancer, both primary and metastatic (10, 41). Another limitation included small sample size of patients because we focused on the high-risk prostate cancer patients who had non-regional LNMs in this project, which will be helpful for clinical decision making.

In conclusion, our study demonstrated that the clinical nomograms have the potential to predict non-regional LNMs. Our findings also demonstrate the proportion of non-regional metastases in the initial staging by ^68^Ga-PSMA PET/CT and demonstrate how this information may impact clinical decision-making in high-risk treatment-naïve PC patients, especially in those with non-regional LNMs. In comparison to the current standards for PLND, our study revealed that non-regional LNMs can be observed in more than one-third of patients, and ^68^Ga-PSMA PET/CT has the potential to add additional information to existing nomograms-based clinical decision-making in more than two-thirds of the high-risk PC patients. We focused on that a threshold can be set for current clinical PLND-validated nomograms to predict extrareolar LN metastases with an AUC accuracy of about 80% after optimizing the simple nomograms which may help to improve the efficiency for PC therapy significantly in clinical practice. If validated in a larger prospective study, ^68^Ga-PSMA PET/CT, as well as clinical nomograms, can be used to exclude patients with non-regional LNMs before PLND with the potential to more accurately identify the appropriate treatment modality for patients.

## Data Availability Statement 

The datasets presented in this article are not readily available because N/A. Requests to access the datasets should be directed to qinwj@fmmu.edu.cn.

## Ethics Statement 

The studies involving human participants were reviewed and approved by the Ethics Committee of Fourth Military Medical University. The patients/participants provided their written informed consent to participate in this study. Written informed consent was obtained from the individual(s) for the publication of any potentially identifiable images or data included in this article.

## Author Contributions

All authors listed have made a substantial, direct, and intellectual contribution to the work and approved it for publication.

## Funding

This study is funded by the National Natural Science Foundation of China (grant nos. 81772734, 81971646, 91959208, 81871379 and 81372748), Lynn Sage cancer research OncoSET program, Innovation Capability Support Program of Shaanxi (grant nos. 2020PT-021 and 2021TD-39) and Key project of Shaanxi Natural Science Basic Research Program (grant no. 2021JZ-25).

## Conflict of Interest

The authors declare that the research was conducted in the absence of any commercial or financial relationships that could be construed as a potential conflict of interest.

## Abbreviations

LNMs, lymph node metastases; PC, prostate cancer; MDT, multidisciplinary cancer therapy; PSMA, prostate specific membrane antigen; PET/CT, positron emission tomography computed tomography; PLND, pelvic lymph node dissection; ePLND, extend PLND; sPLND, sentinel PLND; CT, computed tomography; mpMRI, multiparametric magnetic resonance imaging; DWI, diffusion-weighted imaging; USPIO, ultra-small paramagnetic iron oxide particles; tPSA, total prostate specific antigen; GS, Gleason score; ADT, androgen deprivation treatment; RP, radical prostatectomy; RT, radiotherapy; IHC, immunohistochemistry; IF, immunofluorescence; l.n., lymph nodes; BMs, bone metastases; IGRT, image-guided radiotherapy; SUV_max_, Maximum Standardized Uptake Values; HE, hematoxylin-eosin; RP, radical prostatectomy; TRUS, transrectal ultrasound; AUC, Area under curve.
