# Supplementary material for: The Establishment of New Thresholds for PLND-Validated Clinical Nomograms to Predict Non-Regional Lymph Node Metastases: Using 68Ga-PSMA PET/CT as References
Source: Front Oncol. 2021 Apr 15;11:658669. doi: 10.3389/fonc.2021.658669 (PMC8082014; doi:10.3389/fonc.2021.658669)
Supplement: Supplementary file 1 [file DataSheet_1.docx]

**2. Supplementary materials and methods**

**2.3 Immunohistochemistry (IHC) staining**

Paraffin-embedded primary PC tissue and resected lymph node tissue wax blocks were sliced into 4-μm-thick tissue pieces and then placed on glass sides. Slides were subjected to immunohistochemical (IHC) staining to evaluate PSMA expression. Briefly, slides were deparaffinized in xylene and rehydrated through a series of ethanol solutions; then, antigen retrieval was performed in boiling EDTA buffer (1.27 mmol/L ethylenediamine tetraacetic acid/1 mmol/L Tris, pH 9.0) for 15 minutes. After inactivation of endogenous peroxidase with blocking solution (SP KIT-A3, MXB Biotechnologies), three washes in phosphate-buffered saline (PBS) were performed. After blockage with nonimmune goat serum (SP KIT-B3, MXB Biotechnologies) for 20 minutes at room temperature, slides were then incubated with anti-PSMA antibody (1:100, MAB-0575, MXB Biotechnologies) overnight at 4℃. After 3 washes with PBS, specimens were incubated with horseradish peroxidase (HRP)-labeled goat anti-mouse/rabbit IgG (KIT-5030, MXB Biotechnologies) at room temperature for 30 minutes, followed by 3 washes with PBS. The visualization reaction was performed using diaminobenzidine (DAB, DAB-4033, MXB Biotechnologies) and was then stopped after 3 minutes in tap water. After counterstaining with hematoxylin for 5 minutes, slides were then rinsed in water, dehydrated in a series of ethanol with ascending concentration, followed by clearance with xylene, and cover slipped permanently for light microscopy observation. All procedures were performed according to the manufacturer’s instructions. Stained slides were observed by a Nano Zoomer SQ workstation at the 10×40 setting (HAMAMATSU Inc.).

**2.4 Immunofluorescence (IF) staining**

Paraffin-embedded PC tissue slides were prepared as above. Specimens were also deparaffinized, rehydrated, antigen-retrieved, and blocked against endogenous peroxidase and nonspecific protein binding as above. Slides were then incubated with corresponding antibodies in a humidified chamber overnight at 4℃. Slides were then washed with PBS before they were incubated with Cy3-conjugated goat anti-rabbit IgG secondary antibody (GB23303, Wuhan Servicebio Technology, 1:500) and FITC-conjugated goat anti-mouse IgG secondary antibody (GB23301, Wuhan Servicebio Technology, 1:500) for 1 hour at 4℃ in darkness. Slides were then stained with 4ˊ,6-diamidino-2-phenylindole (DAPI) to visualize the nuclei. Finally, slides were sealed by an anti-fluorescence quenching seal tablet (G1401, Wuhan Servicebio Technology) before they were observed and imaged under a laser scanning confocal microscope (NIKON ECLIPSE C1, Nikon Corporation, Japan) and an imaging system (NIKON DS-U3, Nikon Corporation, Japan). Antibodies used in this study included PSMA antibody (MAB-0575, MXB Biotechnologies, 1:50) and P504s antibody (RMA-0546, MXB Biotechnologies, 1:50). P504s, as a widely used biomarker of PC, was used to diagnose nodal malignant cells originated from PC.
