# Supplementary material for: The Establishment of New Thresholds for PLND-Validated Clinical Nomograms to Predict Non-Regional Lymph Node Metastases: Using 68Ga-PSMA PET/CT as References
Source: Front Oncol. 2021 Apr 15;11:658669. doi: 10.3389/fonc.2021.658669 (PMC8082014; doi:10.3389/fonc.2021.658669)
Supplement: Supplementary file 2 [file DataSheet_2.docx]

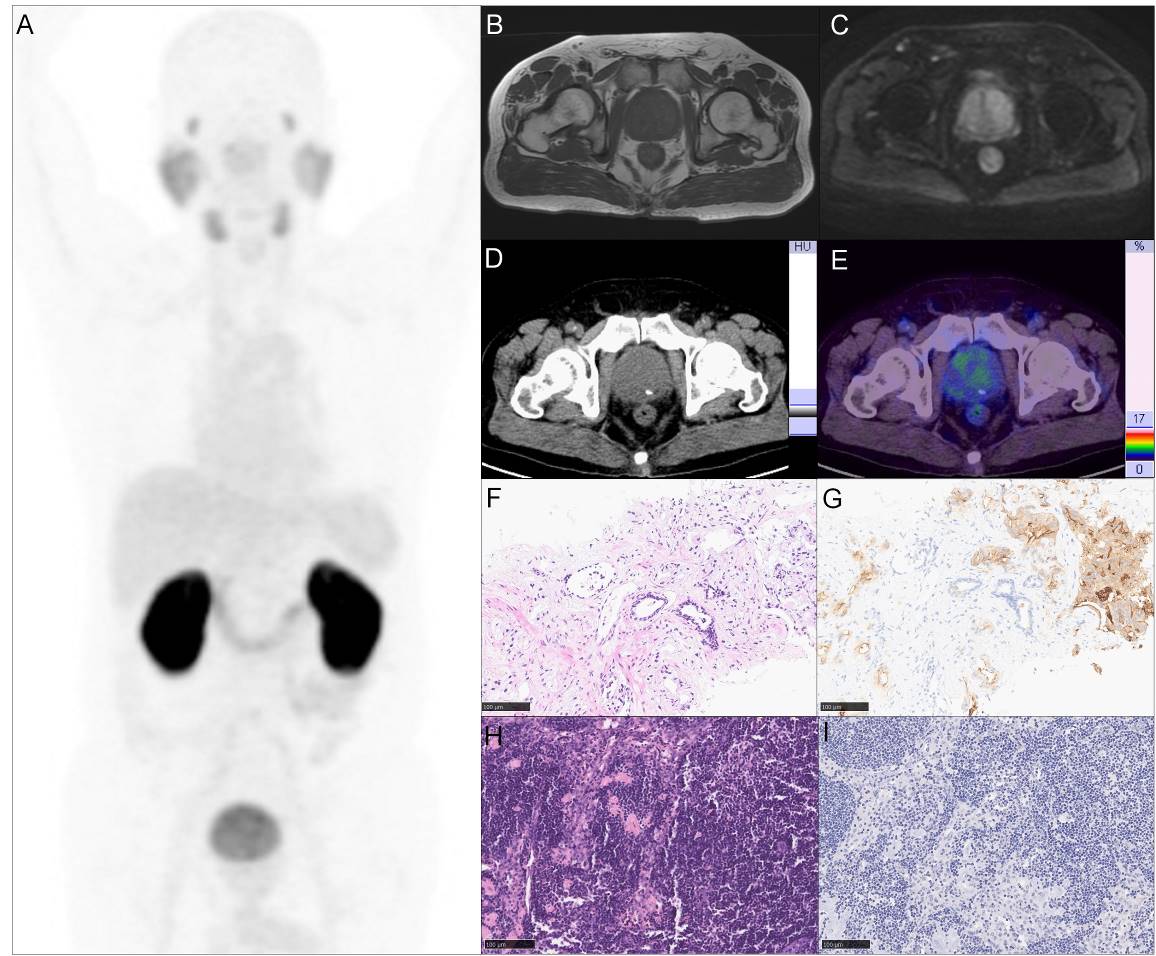


**FIGURE S1.** **Representative PC patient without lymph nodes metastases**

**A, D, E**) ^68^Ga-PSMA PET/CT results of a 71 y/o patient without LNMs (cT2a, Gleason 4+4=8; PSA 9.32 ng/ml; primary tumor, SUV_max_ 3.4). **B, C**) In pelvic mpMRI, there was no obvious abnormal signal on T1W and DWI. **F, H**) HE staining and **G, I**) PSMA IHC staining of **F, G**) primary PC and **H, I**) resected lymph nodes. ^68^Ga-PSMA PET/CT has the potential to reduce ePLND in the patients without LNMs.

**
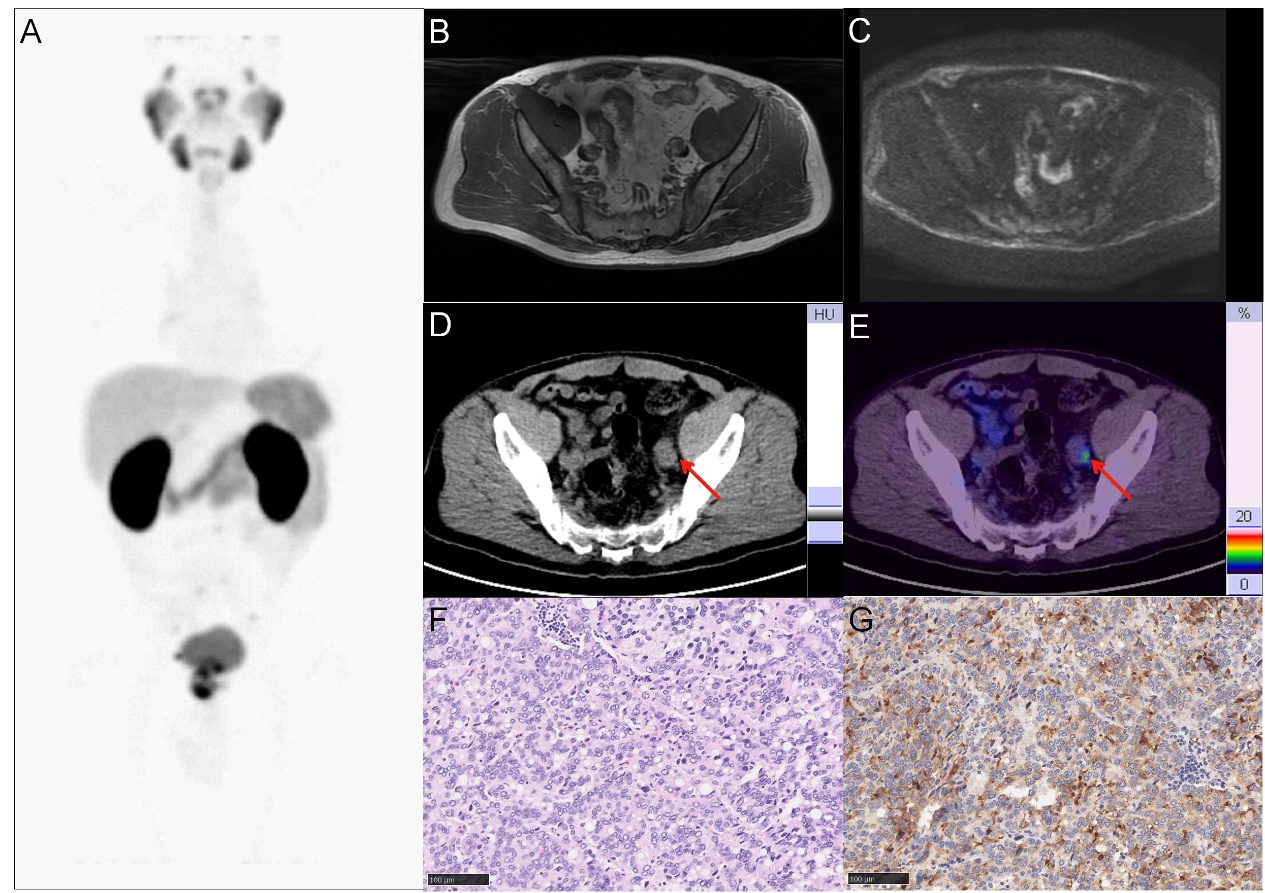
**

**FIGURE S2.** ^68^Ga-PSMA PET can detect lymph node metastases missed by pelvic mpMRI in a representative PC patient. **A, D, E**) ^68^Ga-PSMA PET/CT results of a 65 y/o patient with lymph node metastases (cT3b, Gleason 4+4=8; PSA 142.08ng/ml; **red arrow**, regional lymph node metastases, SUVmax 2.3); **B, C**) In pelvic mpMRI, there was no obvious abnormal signal on T1W and DWI; **F**) HE staining and **G**) PSMA staining of the resected left para-iliac LNMs.


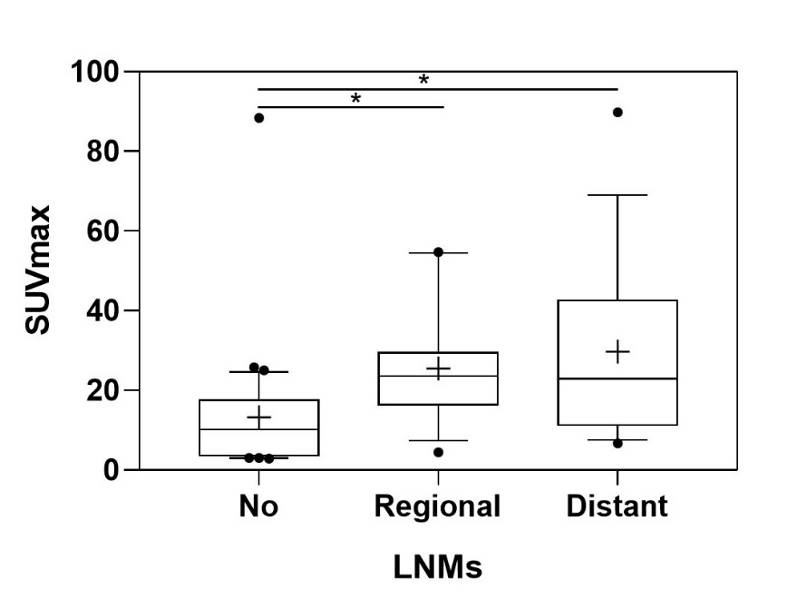


**FIGURE S3.** SUV_max_ comparison in primary PC tumors between the patients with no, regional LNMs and distant LNMs. Vertical boders of box represent 25th and 75th percentiles and middle bar represents median while “+” represents mean. The SUV_max_ in primary PC tumor was higher in the patients with distant LNMs than the patients with regional LNMs, while the patients without LNMs had the lowest SUV_max_. Mann-Whitney U tests revealed significant difference of SUV_max_ in primary tumors between the patients without LNMs and the patients with regional LNMs (*, P=0.002), as well as between the patients without LNMs and the patients with distant LNMs (*, P=0.004).


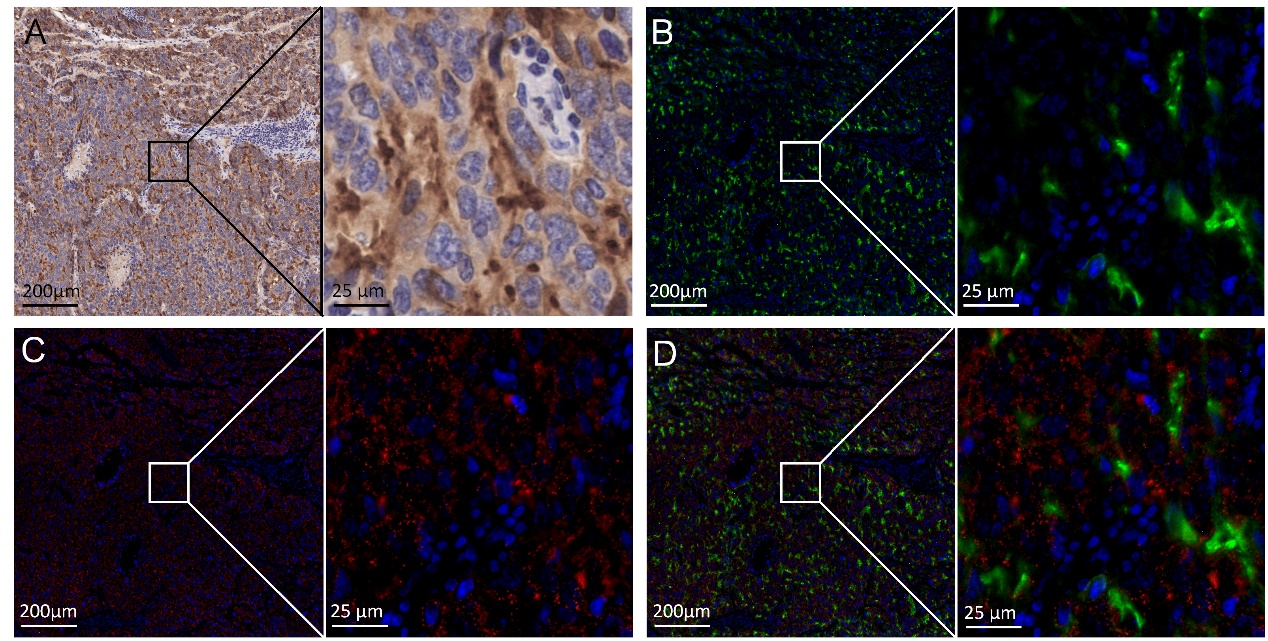


**Figure S4.** Co-expression of PSMA and P504s on LNMs confirmed that the PSMA-avid LNMs originated from PC. **A)** Immunohistochemical staining of PSMA in metastatic lymph nodes (10╳10, 80╳10); **B, C, D)** Immunofluorescence staining of PSMA and P504s in LNMs (Nuclei=blue, P504s=red, PSMA=green, co-expression of P504s and PSMA=yellow, 10╳10, 80╳10).
